# In Situ Industrial Bimetallic Catalyst Characterization using Scanning Transmission Electron Microscopy and X‐ray Absorption Spectroscopy at One Atmosphere and Elevated Temperature

**DOI:** 10.1002/cphc.201700425

**Published:** 2017-07-04

**Authors:** Eric Prestat, Matthew A. Kulzick, Paul J. Dietrich, Mr. Matthew Smith, Mr. Eu‐Pin Tien, M. Grace Burke, Sarah J. Haigh, Nestor J. Zaluzec

**Affiliations:** ^1^ School of Materials University of Manchester Manchester M13 9PL United Kingdom; ^2^ BP Research Center Naperville IL 60563 USA; ^3^ Argonne National Laboratory Photon Sciences Division Argonne IL 60439 USA

**Keywords:** electron microscopy, energy dispersive X-ray spectroscopy, nanoparticles, supported catalysts, X-ray absorption spectroscopy

## Abstract

We have developed a new experimental platform for in situ scanning transmission electron microscope (STEM) energy dispersive X‐ray spectroscopy (EDS) which allows real time, nanoscale, elemental and structural changes to be studied at elevated temperature (up to 1000 °C) and pressure (up to 1 atm). Here we demonstrate the first application of this approach to understand complex structural changes occurring during reduction of a bimetallic catalyst, PdCu supported on TiO_2_, synthesized by wet impregnation. We reveal a heterogeneous evolution of nanoparticle size, distribution, and composition with large differences in reduction behavior for the two metals. We show that the data obtained is complementary to in situ STEM electron energy loss spectroscopy (EELS) and when combined with in situ X‐ray absorption spectroscopy (XAS) allows correlation of bulk chemical state with nanoscale changes in elemental distribution during reduction, facilitating new understanding of the catalytic behavior for this important class of materials.

Supported metal nanoparticles play a vital catalytic role in optimizing the conversion efficiency for a wide range of industrially important chemical reactions. Bimetallic catalysts frequently offer improvements in activity, selectivity, and stability compared to their monometallic equivalents^1^, ^2^, ^3^ for diverse reactions such as catalytic reforming,^4^, ^5^ hydrotreating,^6^, ^7^ and emissions controls.^8^, ^9^ The addition of the secondary element improves functionality but also introduces a greater complexity to the system and often obscures the true nature of the active site, which is dramatically affected by the elemental distribution with individual nanoparticles.^1^, ^2^

The problem is made more complicated as the elemental distributions are frequently inhomogeneous when viewed at the nanoscale and not all particle morphologies present show equal activity for a given reaction.^10^ Additionally, the catalysts are usually required to operate in aggressive chemical environments (elevated temperatures and pressures; liquid environments). The operating environment often leads to unwanted morphological and/or composition changes through surface restructuring or metals leaching,^10^, ^11^ and can degrade catalytic performance.^12^, ^13^ Catalyst degradation is the subject of many books, reviews and more than 20,000 U.S. patents for the period of 1976–2013.^14^ Despite this, little is known about the nanoscale changes that occur when supported bimetallic catalyst materials are exposed to elevated temperature and pressures.

Bulk spectroscopy techniques such as X‐ray absorption spectroscopy (XAS) offer a powerful approach to study averaged changes to oxidation states and local coordination environments which can be tracked as a function of reaction conditions. However, XAS is a bulk technique, and provides an average over all atoms within the beam path (for conventional XAS, the beam size is usually not less than 500 μm), which does not allow for analysis of nanoscale structures. Even the most advanced nanoscale XAS mapping has a minimum achievable spot size for the X‐ray beam of around 15 nm for soft X‐rays (200–2000 eV) and 30–50 nm for hard X‐rays (4000–14 000 eV).^15^ Industrial supported nanoparticle catalysts have typical sizes of ≈1–10 nm, thus limiting nanoscale XAS characterisation to studying ensembles of nanoparticles.

Only transmission or scanning transmission electron microscopy (TEM, STEM) can provide nanoscale structural and elemental information for individual nanoparticles at the required length scale.^16^, ^17^, ^18^ When combined with electron energy loss spectroscopy (EELS) or energy dispersive X‐ray spectroscopy (EDS) this approach can be used to map the elemental distribution or even oxidation states for individual nanoparticles, with a full three dimensional characterisation at nanometre resolution achievable for model catalyst systems and ultra‐high vacuum (UHV) environments.^19^

Although these results are encouraging, recent work has demonstrated that surface structures can vary dramatically in UHV environments from those appropriate to the working conditions of industrial catalysts.^20^ This has led to an explosion of interest in in situ TEM, where materials are studied under more realistic environmental conditions.^21^, ^22^, ^23^ Dedicated environmental transmission electron microscope (ETEM) systems allow for atomic resolution imaging at moderate temperature but are limited to pressures of less than ≈50 mbar.^24^, ^25^, ^26^ The alternative approach is to use specially designed environmental cell (e‐Cell) holders within a conventional (S)TEM instrument.^27^ Here the sample and reaction environment is protected from the instrument vacuum by two thin ≈50 nm SiN windows, allowing imaging at temperatures up to ≈1000 °C and at pressures up to ≈1 bar.^28^

However, both approaches compromise the ultimate (S)TEM imaging resolution and spectroscopy capabilities compared to UHV imaging.

(S)TEM‐EDS allows microanalysis of many elements simultaneously without prior knowledge of the sample composition. However, the application of EDS to in situ experiments has long been restricted by the geometry of the e‐Cells (because the penumbra of the specimen holder prevent X‐rays generated at the sample from reaching the detector). (S)TEM‐EELS spectroscopy is generally superior to EDS for light element analysis although not all elements relevant to catalysis are suitable for EELS analysis. Unfortunately EELS is strongly influenced by the unavoidable effects of multiple scattering;^28^ samples should typically be less than ≈100 nm thick in order to minimise multiple scattering and optimise signal‐to‐noise ratios. The two e‐Cell windows have a combined thickness of 80–100 nm even before the gas and specimen are considered. The concomitant multiple scattering which ensues can prevent even light elements from being resolved. As a consequence, most in situ (S)TEM experiments of supported catalysts to date have not used the spectroscopy capabilities of the instrument but have focused on monometallic or model catalyst systems.^23^, ^24^, ^25^ In particular, those with a high atomic number difference between the support and the nanoparticle so that imaging alone can distinguish morphological changes. In this study, we demonstrate how in situ STEM characterisation can be extended to provide new insights into chemical and morphological changes for supported bimetallic nanoparticle catalysts.

Here we have employed a customised e‐Cell in situ holder with the latest high efficiency EDS detectors and aberration corrected STEM instrumentation to overcome previous limitations associated with in situ STEM‐EDS imaging and elemental mapping at elevated temperature and pressures. We use this approach to investigate industrially relevant supported catalysts using a titania supported PdCu bimetallic catalyst synthesized by wet impregnation. Recent studies have demonstrated that the PdCu bimetallic has improved rate and selectivity for oxygenate coupling reactions than either metal alone, and that the structure of the bimetallic particles is critical for the observed improvement in performance.^29^, ^30^ STEM imaging and elemental mapping of the calcined material in vacuum reveals that it is typical of many industrial catalysts: it has a low metal loading (1.5 wt.% Pd, 0.3 wt.% Cu) heterogeneously dispersed on an irregular, high Z, macroscopic support.

Compositions such as this are difficult to understand without the full capabilities of the modern STEM. Simple high angle annular dark field (HAADF) atomic number contrast (Z‐contrast) images acquired under ex situ vacuum conditions can distinguish the titania support (grey) from the higher intensity metal nanoparticles but the relative locations of Pd and Cu cannot be distinguished (Figure 1 a). Applying STEM‐EDS reveals a heterogeneous metal distribution with Pd present in large particles (ca. 5–10 nm) and Cu distributed more uniformly on the support in clusters (ca. 1 nm or smaller) (Figure 1 b and c). In comparison, the simultaneously acquired EELS spectral signature for Cu and Pd is much poorer due to the combination of low metal concentrations and high Z support (Figure 1 d and SI). This is a common problem in industrial catalysts which often seek to minimise metal loadings to reduce cost.

**Figure 1:**
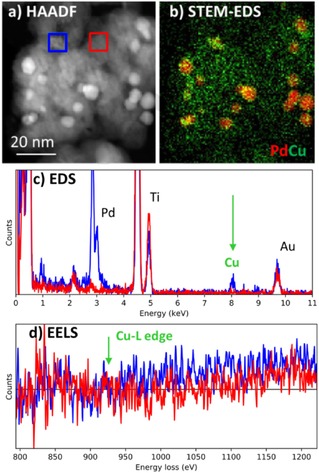
Characterization of calcined PdCu titania supported catalyst in vacuum. a) HAADF image where intensity differentiates the denser Pd particles from the titania support. b) STEM‐EDS elemental mapping which demonstrates that the visible nanoparticles are principally Pd (red) while Cu (green) is more uniformly distributed on the titania support. A comparison of the simultaneously acquired spectral signatures (from the red and blue square regions on (a)) is shown for c) EDS and d) EELS respectively. The EELS signature for Cu is below the signal to noise demonstrating the difficulty of using EELS for mapping of transition metal catalysts at low loadings on a relatively thick support.

X‐ray absorption near edge fine structure (XANES) measurements obtained using an in situ experimental system at the ANL APS synchrotron provide complementary information on the bulk changes to chemical state that occur in the catalyst when heated in reducing conditions (Figure 2 and Supporting Information, SI). After initial calcination, both Pd and Cu are present as nanoscale oxides (for discussion see the SI). Upon heating in H_2_, the Pd reduced immediately to metallic Pd and does not show any change on further heating. Cu remained ≈30 % oxidised at 250 °C and required a 550 °C heat treatment to fully reduce the oxide (confirmed by a lack of Cu‐O coordination in the extended X‐ray absorption fine structure (EXAFS), see the SI). The shape of the Cu XANES differs from the bulk metal reference, which is consistent with having small metal clusters or CuPd alloy nanoparticles. An accurate fit to the EXAFS data required inclusion of both Pd‐Cu and Cu‐Pd scattering pathways and resulted in total coordination numbers lower than the bulk (CN_Pd‐X_<12, CN_Cu‐X_<12), consistent with the presence of some fraction of bimetallic CuPd alloy nanoparticles (for full discussion see SI, Table S.1).

**Figure 2:**
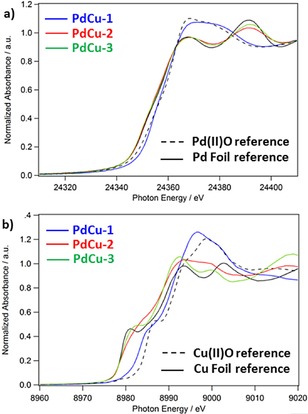
XANES analysis of a) Pd K and b) Cu K reveals bulk changes in the chemical state of the PdCu samples where PdCu‐1 (blue) is after initial calcination at 500 °C in flowing air for 4 hours, while PdCu‐2, PdCu‐3 samples are the same material after reduction in H_2_ for 45 minutes at 250 °C and 550 °C respectively. Comparison to standard references reveals that for PdCu‐1 both Pd and Cu are present as oxides, while in PdCu‐2 and PdCu‐3, Pd metal and Cu metal clusters are present.

To fully interpret how this reduction behavior might affect the catalytic performance in such a complex system requires consideration of the relative interactions of Pd and Cu at the nanometre scale. Only in situ STEM‐EDS results can provide this essential information (Figure 3).

**Figure 3:**
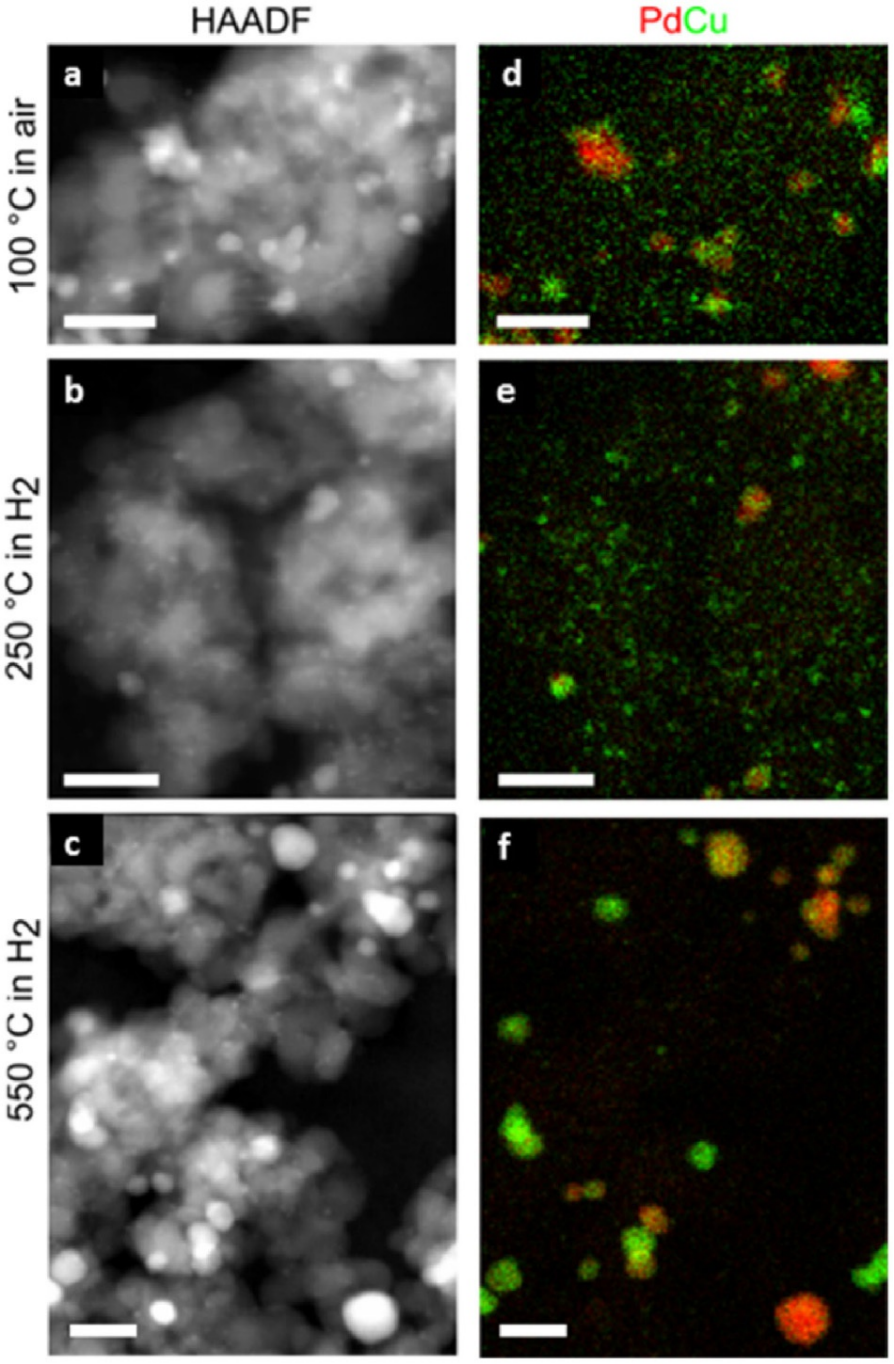
In situ STEM‐EDS analysis of the change in Pd and Cu elemental distribution on reduction in H_2_. Conditions within the e‐Cell are given to the left of the images. Pressures are ≈1 atm. (a‐c) show HAADF STEM images while (c‐f) show elemental maps extracted from EDS spectrum images (Pd, red; Cu, green). Scale bars are all 25 nm. Further data available in the SI.

STEM‐EDS observation of the sample in air at 100 °C shows a similar metal distribution as was observed for the sample in vacuum ex situ (Figure 1). Heating to 250 °C under 1 atm of hydrogen for ≈1 hour in the e‐Cell does not cause observable changes to the HAADF images but results in additional clustering of the Cu distribution on the titania support (Figure 3 d,e,f). It is interesting to note that the Pd is present as PdO in Figure 3 a,d but Pd metal in Figure 3 b,e (as evidenced by the XANES), although this has not produced observable morphological changes to the individual particles. In contrast only ≈70 % of the Cu has reduced to Cu metal. We speculate that Cu that is closely situated to the Pd may be more easily reduced. Subsequent heating to 550 °C under 1 atm of hydrogen for ≈1 hour in the e‐Cell, results in a dramatic decrease in the Cu signal detected within the titania support (see SI, Figure S.9). In parallel there is agglomeration of Cu to Pd nanoparticles (Figure 4), while pure Cu particles are found in areas of the support that appear bare before reduction. Since different nanoparticle structures may have varying catalytic activity,^13^ an understanding of the different particle types observed gives additional insight into catalytic performance. This is particularly important in cases where particle morphology is affected by the environment.^20^, ^21^, ^22^ In systems such as these, in situ high spatial resolution EDS is required to observe the true nanostructures and their compositional distribution as shown in Figure 3 f.

**Figure 4:**
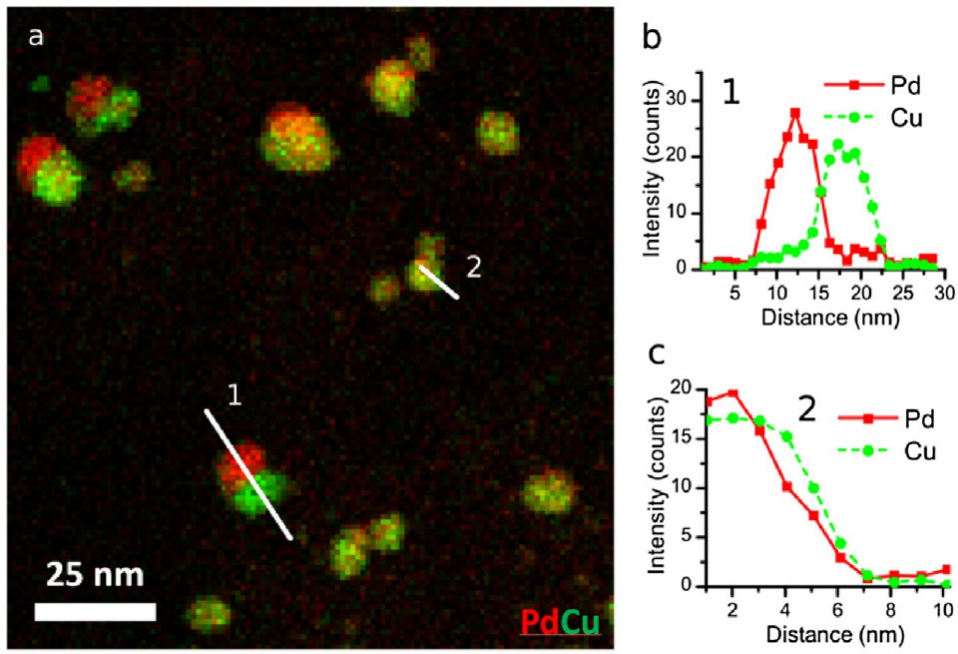
a) Composite STEM‐EDS elemental map for Cu (green) and Pd (red) showing the formation of Janus NPs as well as Cu‐surface‐rich PdCu nanoparticles. b,c) Spectral line scans showing the different compositional distributions (1‐Janus and 2‐Cu‐surface‐enrichment).

Nanoparticle size analysis from multiple STEM‐EDS elemental maps (see SI, Figure S.10) reveals a bimodal distribution in the starting material (Figure 3 d) with a small particles being ≈1.5 nm in diameter and larger particles with diameters of ≈5.5 nm. After reducing at 250 °C both particle types increase in size slightly to ≈2 nm and ≈6 nm, respectively. After further reduction at 550 °C the smaller particles remain around 2 nm in diameter but the larger particles have considerably increased in size to >8 nm. Ostwald ripening at elevated temperature may increase the size of larger particles relative to smaller ones but further metal reduction will also affect the measured particle size distributions.

Closer analysis of the larger metal nanoparticles (5–20 nm in diameter) reveals a wide variety of morphologies (Figure 4). In support regions neighbouring the original Pd‐rich nanoparticles the Cu shows a tendency to form Pd‐core‐Cu‐shell (Figure 4 c) or Janus particles (Figure 4 b). The reverse structure (Cu‐core‐Pd‐shell) was not observed suggesting that Cu coats the pre‐existing Pd nanoparticles during reduction, consistent with the slight increase in mean particle size which we measure (SI).

This investigation has confirmed that combining in situ STEM‐EDS with in situ XANES provides a more complete picture of the formation of the catalyst than either technique can achieve alone. In isolation bulk data such as XANES or temperature programmed reduction (SI, Figure S1) demonstrate the stepped reduction behavior and changes in oxidation state but STEM‐EDS is required to determine the morphology of nanostructures, where more than one metal is present. The lack of significant particle movement in the reduction up to 250 °C despite the complete reduction of Pd and partial reduction of Cu, together with the change in size distribution is strong circumstantial evidence for Pd assisting in the Cu reduction. By 550 °C, the Cu reduction is complete and the remaining Cu becomes mobile, and reduces/agglomerates on the PdCu bimetallic clusters formed previously during the low temperature reduction. Observing the localized behavior demonstrates directly important steps in the formation of industrial bimetallic nanoparticle catalysts.

In conclusion, in addition to resolving the nature of the nanoscale evolution of our PdCu catalyst, we have demonstrated that in situ STEM‐EDS at atmospheric pressures and elevated temperatures has significant potential for the catalysis community; being complementary to in situ XAS measurements and superior to STEM‐EELS for some types of catalytic systems. The ability to image catalyst systems under catalytically relevant conditions that may be replicated in reactor systems can lead to new understanding of structure/function relationships. Performing STEM‐EDS in an e‐Cell holder system has the advantage that they are compatible with a variety of electron microscopes making them potentially more widely accessible to the scientific community. This combination of in situ nanoscale elemental analysis, platform adaptability, and range of operating pressure and temperature makes this technology a significant new resource for the design and understanding of catalyst systems.

## Experimental Section

### Catalyst Synthesis

The PdCu catalyst was prepared by incipient wetness co‐impregnation of solutions of Pd(NO_3_)_2_ and Cu(NO_3_)_2_ dissolved in deionized (DI) water to a sample titanium dioxide. The metal loadings in solution were targeted to give a total metal loading of 2 wt.% with a Pd:Cu molar ratio of 3:1 (1.7 wt.% Pd, 0.3 wt.% Cu). The catalyst was air dried at 120 °C overnight and then calcined at 500 °C in flowing air for 4 hours.

### Temperature Programmed Reduction

Temperature programmed reduction (TPR) experiments were conducted on a Micromeritics 2920 analyzer fitted with a Cryocooler II cryogenic sampler cooler and a Pfeiffer Thermostar Mass spectrometer. Samples were preconditioned by heating to 200 °C at 20 °C min^−1^ and holding for two hours under 10 mL min^−1^ flowing argon. Samples were then cooled to −50 °C using the Cryocooler II, exposed to 10 % hydrogen/argon flowing at 10 mL min^−1^, and held for fifteen minutes at low temperature before initiating the TPR experiment. In the TPR, a ramp rate of 5 °C min^−1^ to a final temperature of 500 °C and a final hold of one hour was used. Hydrogen uptake and product analysis were measured by a mass spectrometer (monitoring mass channels 2 and 18) and a thermal conductivity detector (TCD). In some experiments a dry ice trap was used to remove water and other condensable components from the effluent gas stream to better observe hydrogen consumption in the TCD trace.

### X‐ray Absorption Spectroscopy

X‐ray absorption spectroscopy experiments were conducted at the Sector 10 Materials Research Collaborative Access Team (MRCAT) insertion device (ID) and bending magnet (BM) beam lines at the Advanced Photon Source (APS) at Argonne National Laboratory (ANL). Catalyst samples were measured at both the Pd K (24350 eV) and Cu K (8979 eV) X‐ray absorption edges after in situ gas treatments. Pd K edge experiments were conducted in transmission mode with an in situ gas cell. The samples were loaded into a 6‐well sample holder and pressed into self‐supporting wafers. Sample loading was 50 mg, corresponding to a total absorbance (μx) of 1.0 and an edge step (Δμx) of 0.3. The samples were loaded into a 1“ OD quartz tube, with Ultra‐torr fittings and welded ball valves at either end to control the gas atmosphere. Cu K edge experiments were conducted in fluorescence mode with a 4 element solid state Si drift detector (SII Nanotechnology, Model Vortex ME‐4). The sample was pressed into a thin wafer in a custom sample holder angled at 45 degrees for maximum fluorescence yield and was analyzed while inside a special in situ fluorescence cell, which allowed for a sealed gas atmosphere. Catalyst samples were treated in their respective in situ XAS cells under a 3.5 % H_2_/He gas mixture and were measured as received, and then after treatments at room temperature and 200, 250, 350, and 550 °C. For the elevated temperature studies, samples were heated to the target temperature and held for 45 min, purged with He at the reduction temperature for 15 min, and then cooled to RT under flowing He. Measurements were conducted in a static He atmosphere at room temperature.

### Analytical Electron Microscopy

Analytical electron microscopy (AEM) spectrum imaging measurements were carried out using a FEI Titan G2 80–200 (S)TEM ChemiSTEM^TM^ as well as an FEI Tecnai F20 (S)TEM instrument both operating at 200 keV at the University of Manchester and Argonne National Laboratory, respectively. The Titan instrument was equipped with the FEI SuperX Quad SDD array system (≈0.7 sR collection angle) with Bruker Esprit analysis package, while the Tecnai F20 was equipped a EDAX Apollo XLT SDD (≈0.3 sR collection angle) with TEAM data acquisition software. STEM HAADF images were recorded using FEI TIA software on both instruments while spectrum images were recorded using Gatan DigitalMicrograph, Bruker Esprit and EDAX TEAM hyperspectral software on the Titan and Tecnai instruments, respectively. Post processing of STEM‐EDS hyperspectral images was carried out using DigitalMicrograph, Esprit, TEAM as well as customized software programs developed by the authors. The gaseous e‐Cell system used in the AEM instruments for this study was a Protochips Atmosphere holder having customized low penumbra geometry beryllium lid. The e‐Cell MEMS chips used to create the operando environment were of a pair of 300 μm thick Si wafers; with each having a lithographically fabricated, 3000×300 μm, electron transparent SiNx window. The upper chip allows heating and has a SiNx window thickness of 30 nm, while the lower window is 50 nm thick. Spacers deposited onto the chips created a nominal vertical separation between the windows of approximately 5 μm. During AEM measurements the e‐Cell was completely filled with either pure H_2_ gas or room air at nominally 1 bar pressure while the temperature was controlled by on‐chip pre‐calibrated heater elements controlled by an external computer system.

Catalyst specimens from the same batch of material used for XAS studies were crushed and dispersed in methanol or ethanol and then drop cast onto the atmosphere side of the electron entrance of plasma cleaned SiNx windows and allowed to dry in a clean ambient temperature petri dish. Finally the prepared e‐Cell was sealed as per manufacturer's specification and specimens were allowed to stabilize at room temperature in the documented gaseous media prior to all measurements. To ensure electron beam artefacts were not contributing to the results, images of selected regions of interest were acquired pre and post thermal treatments. As a further check, post thermal treatment, different sample areas which had not previously been subjected to electron beam irradiation but which had experienced the same in situ treatments were also measured to insure that the results reported herein are representative and reproducible. Images were captured using the same treatment protocols as the XAS. Following temperature stabilization, the samples were allowed to sit for 1 hour under the gas environment before imaging and mapping were performed to remove any potential variances due to time.

## Conflict of interest

*The authors declare no conflict of interest*.

## Supporting information

As a service to our authors and readers, this journal provides supporting information supplied by the authors. Such materials are peer reviewed and may be re‐organized for online delivery, but are not copy‐edited or typeset. Technical support issues arising from supporting information (other than missing files) should be addressed to the authors.

Supplementary
