# Supplementary material for: In Situ Industrial Bimetallic Catalyst Characterization using Scanning Transmission Electron Microscopy and X‐ray Absorption Spectroscopy at One Atmosphere and Elevated Temperature
Source: Chemphyschem. 2017 Jul 4;18(16):2151–6. doi: 10.1002/cphc.201700425 (PMC5577507; doi:10.1002/cphc.201700425)
Supplement: Supplementary file 1 — Supplementary [file CPHC-18-2151-s001.pdf]

CHEMPHYSCHEM

Supporting Information

In Situ Industrial Bimetallic Catalyst Characterization using Scanning Transmission Electron Microscopy and X-ray Absorption Spectroscopy at One Atmosphere and Elevated Temperature

Eric Prestat,[a] Matthew A. Kulzick,[b] Paul J. Dietrich,[b] Mr. Matthew Smith,[a] Mr. Eu-Pin Tien,[a] M. Grace Burke,[a] Sarah J. Haigh,*[a] and Nestor J. Zaluzec*[a, c]

cphc_201700425_sm_miscellaneous_information.pdf

S1) TEMPERATURE PROGRAMMED REDUCTION

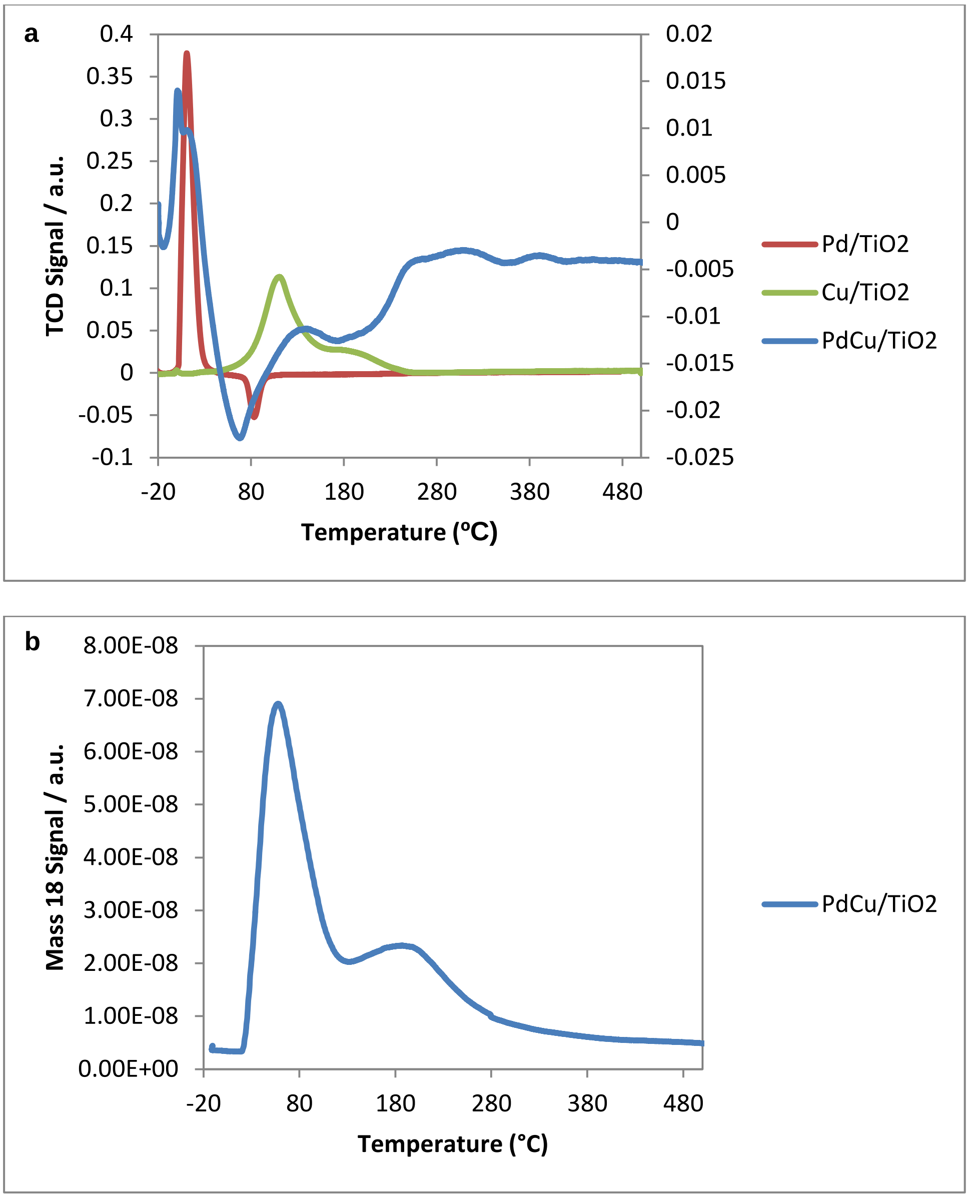

Figure S.1. a) Temperature programmed reduction of our PdCu/TiO_2 catalyst compared to reference materials (monometallic Pd on TiO_2, Cu on TiO_2). The TCD signals are inverted so that peaks correspond to H2 consumption and valleys correspond to H_2 release; b) Mass 18 mass spectrometry signal for the TPR of PdCu/TiO_2.

The temperature programmed reduction (TPR) measurements obtained for the PdCu/TiO_2 catalyst is shown in Figure S.1. The TPR peaks can be assigned based on comparison to literature for related Pd and Cu systems [1, 2]. At low temperature (< 40°C), a series of peaks consistent with the H_2 reduction of Pd are observed on both the PdCu/TiO_2 and Pd/TiO_2. At temperatures between 80 and 280°C, the monometallic Cu/TiO_2 is observed to reduce in a stepwise manner (CuO -> Cu_2O -> Cu metal), suggesting some form of isolated copper species on the TiO_2 surface in the bimetallic catalyst.

S2) X-RAY ABSORPTION SPECTROSCOPY

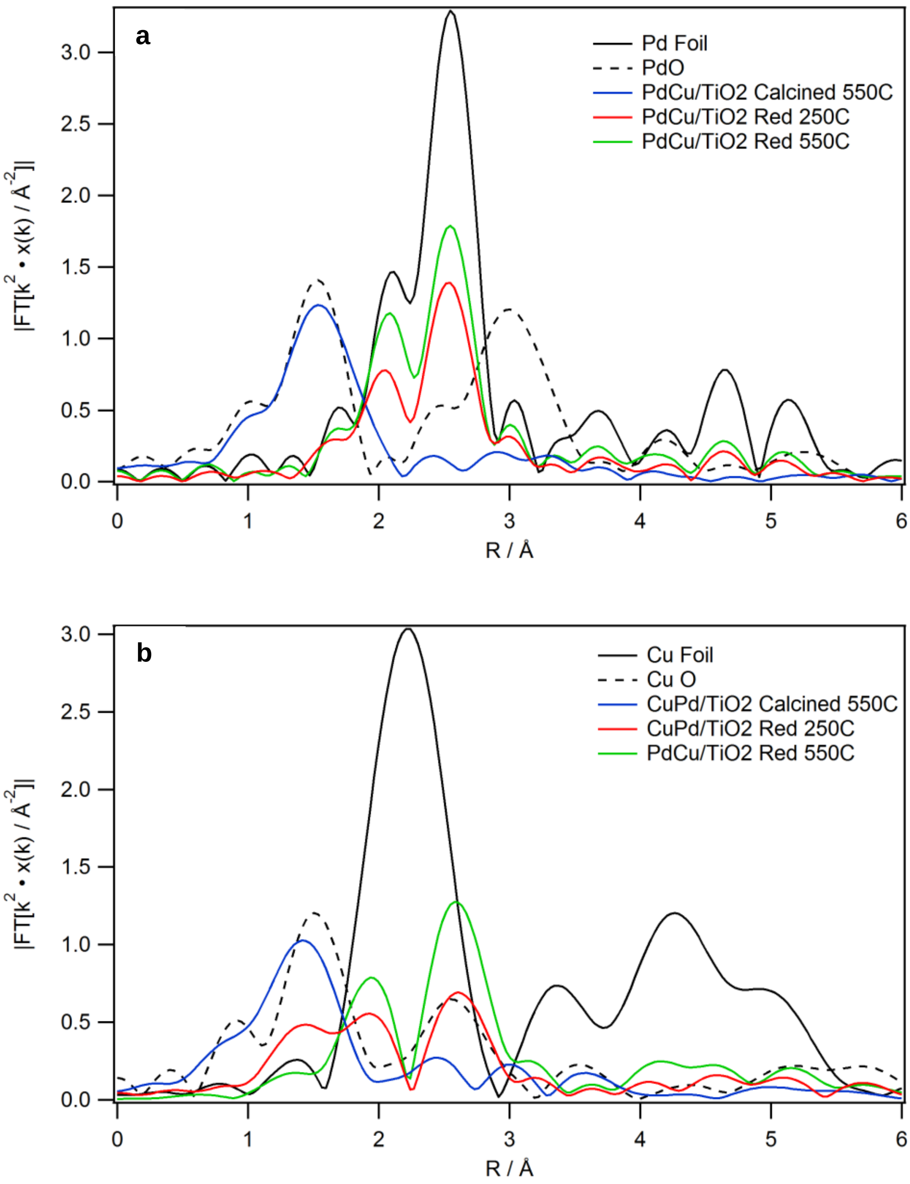

Figure S.2. Magnitude of the Fourier transform of the k^2-weighted extended X-ray absorption fine structure (EXAFS, χ(k)), generating a pseudo-radial distribution function (R), for the PdCu/TiO_2 catalyst. a) Pd K-edge data taken after 550°C calcination, 250ºC reduction, and 550ºC reduction compared to Pd foil and PdO bulk references; b) the Cu K-edge data taken after 550ºC calcination, 250 ºC reduction, and 550ºC reduction compared to CuO and Cu foil bulk references.

Table S.1. EXAFS fitting parameters for the $PdCu/TiO_2$ catalyst at both the Pd K and Cu K edges.

Red. Temp	Edge	Edge Energy	Absorber-Backscatter	N	R / Å	Δσ2/ 10-3 Å2	ΔE0 / eV	N Total	%MeOx
None	Pd K	24355.3	Pd-O	4	2.05	0.005	3.3	--	100%
	Cu K	8983.1	Cu-O	3.3	1.93	0.006	2.6	--	82%
250°C	Pd K	24350	Pd-Pd	6.2	2.71	0.001	-2.2	7.4	0%
			Pd-Cu	1.2	2.67	0.001	-2.2		
	Cu K	8979	Cu-Cu	0.7	2.57	0.002	-1.1	6.1	31%
			Cu-Pd	3.5	2.67	0.002	-1.1		
			Cu-O	1.2	1.96	0.002	6.9		
550°C	Pd K	24350	Pd-Pd	7.6	2.71	0.001	0.8	9.8	0%
			Pd-Cu	2.3	2.63	0.001	-0.8		
	Cu K	8979	Cu-Cu	1.7	2.57	0.002	-2.6	8.5	0%
			Cu-Pd	6.8	2.63	0.002	-2.6		

Figure S.2 presents the magnitude of the Fourier transform of the k^2-weighted extended X-ray absorption fine structure (EXAFS, χ(k)) for the $PdCu/TiO_2$ catalyst after reduction at the temperatures specified in this study. This further supports the reduction data presented in the main paper (Figure 2). Specifically, because the shape of the XAS edge was so different from that of the reference Cu foil, this Fourier transform data was used to confirm complete reduction of the Cu species in the catalyst. This was done by comparing the Cu-O scattering region (peaks between R = 1 and 2 Å) for our sample to the CuO reference. We observe no Cu-O scattering in the sample reduced at 550°C, indicating complete reduction of the Cu. The extent of reduction may also be estimated from the estimated Cu-O coordination number (N). Copper oxide species in the +2 oxidation will always have 4 Cu-O single bonds (less if double bonds are present) – in general, unlike metal nanoparticles, oxides will not have fractional coordination numbers. Thus, a fractional Cu-O coordination number in the fitting is indicative of a fraction of CuO species. The fraction of oxidized species can be estimated using the following relationship:

$$N_{Cu-O,observed} = 4 * \%_{Cu-O}$$

Thus, by dividing the calculated N_{Cu-O} by 4, the fraction of CuO can be estimated from the EXAFS.

The EXAFS fits in Table S.1 are evidence of the formation of PdCu alloy nanoparticles. Fitting after reduction at 250 and 550 °C showed evidence of both Cu-Pd and Pd-Cu scattering pathways, consistent with the bimetallic nature of the material even after incomplete reduction of the Cu. The The shapes of the XANES edges for both the Pd K (Figure 2a) and Cu K (Figure 2b) have slightly different shapes compared to bulk oxides, which is consistent with the nanoparticulate nature of the samples [3].

S3) EX SITU STEM-EDS ANALYSIS OF PdCu/TiO_2 CATALYSTS

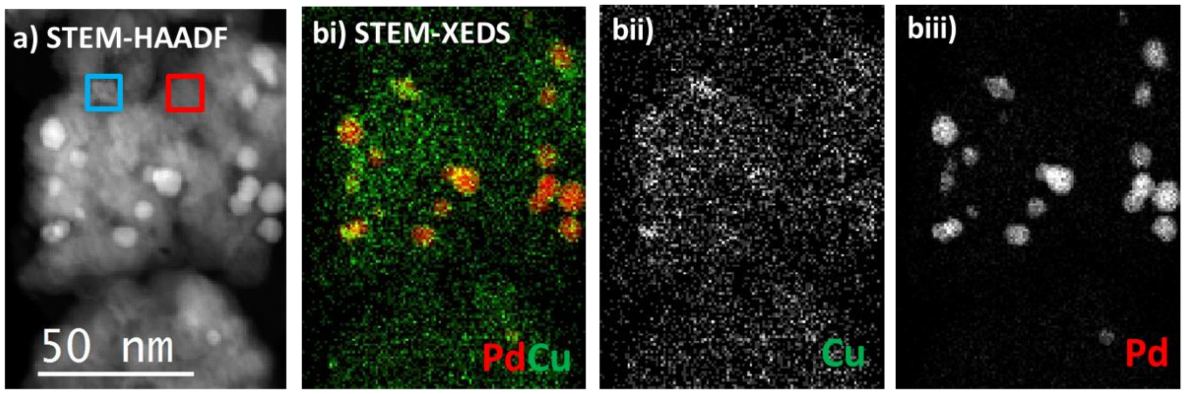

Figure S.3. Representative ex situ a) HAADF image, b) Pd and Cu STEM-EDS elemental maps for calcined PdCu/TiO_2 starting material. (bi) composite Pd-Cu image. (bii) Cu elemental map and (biii) Pd elemental map from which (bi) was formed.

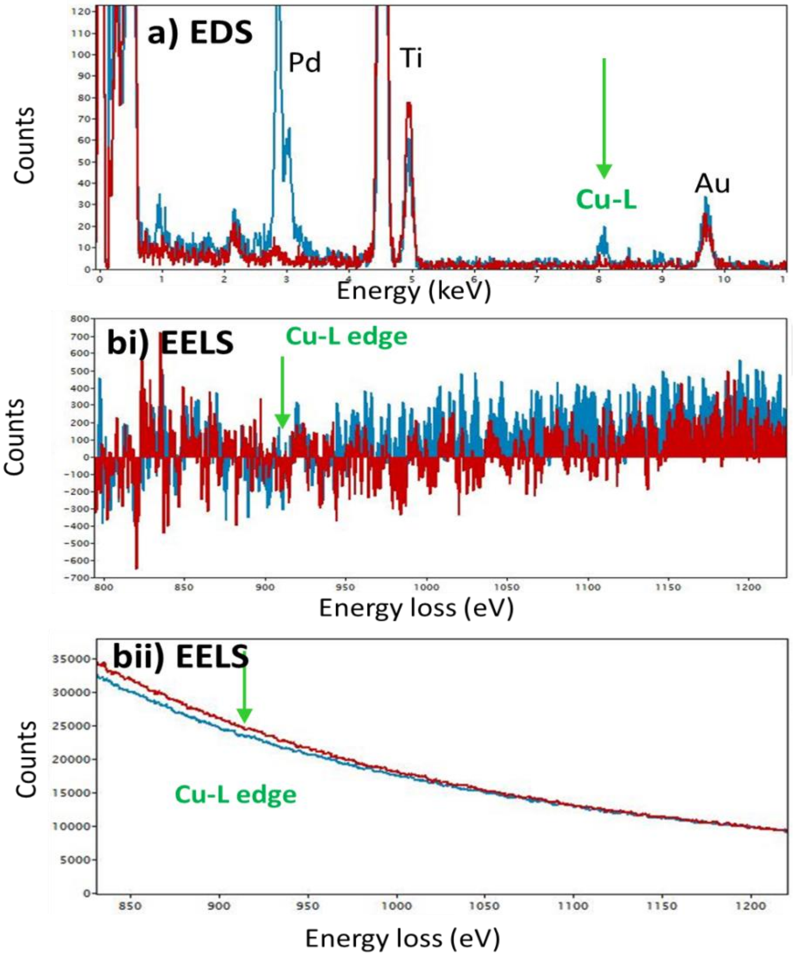

Figure S.4. Ex situ simultaneously acquired (a) STEM-EDS and (b) STEM-EELS data from the red and blue square regions shown in Figure S.3a. A higher Cu signal is clearly visible in the blue region using STEM-EDS but STEM-EELS shows no differences. (bi) EELS data after background subtraction and (bii) raw EELS data.

S4) IN SITU STEM-EDS ANALYSIS OF PdCu/TiO_2 CATALYSTS

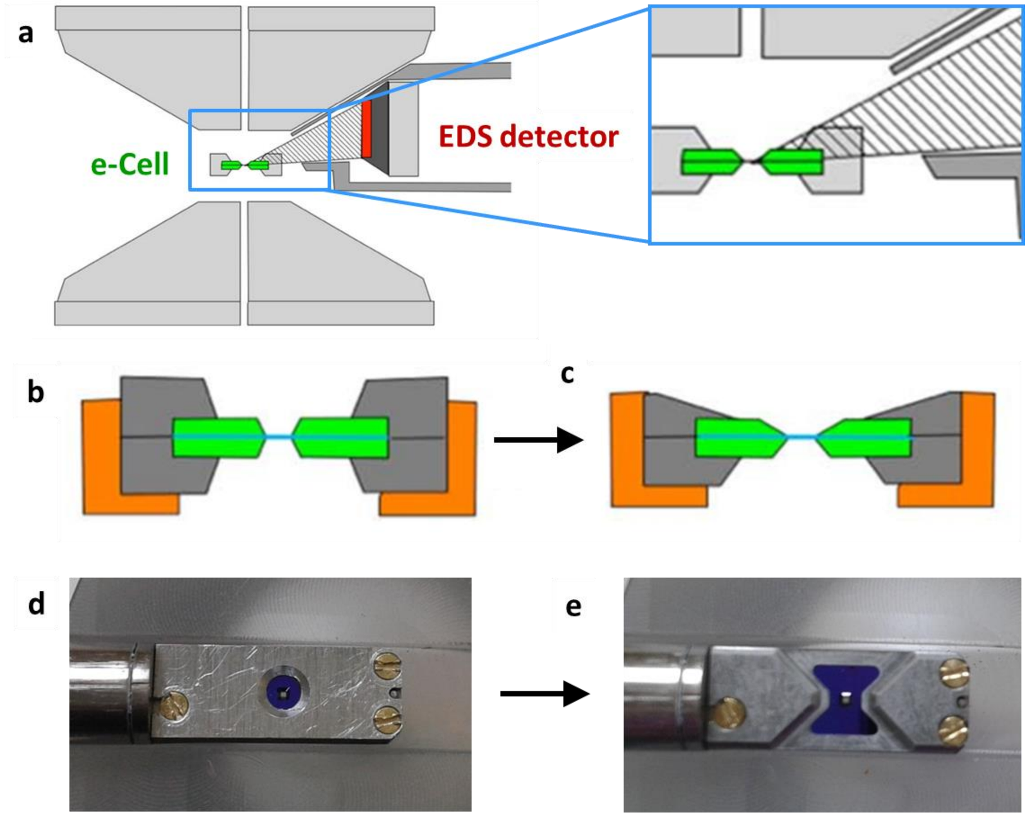

Figure S.5. **Acquiring STEM-EDS data in situ**. (a) Schematic diagram of the STEM pole piece/sample holder geometry illustrating the difficulties associated with acquiring EDS signal from a traditional e-Cell in situ holder. Schematic cross sections and plan view optical images of the holder tip are compared for the original e-Cell holder (b, d) and after modification of the top plate to allow STEM-EDS analysis (c, e). For further details see Zaluzec *et al.* Microsc. Microanal. (2014) 20 p. 323

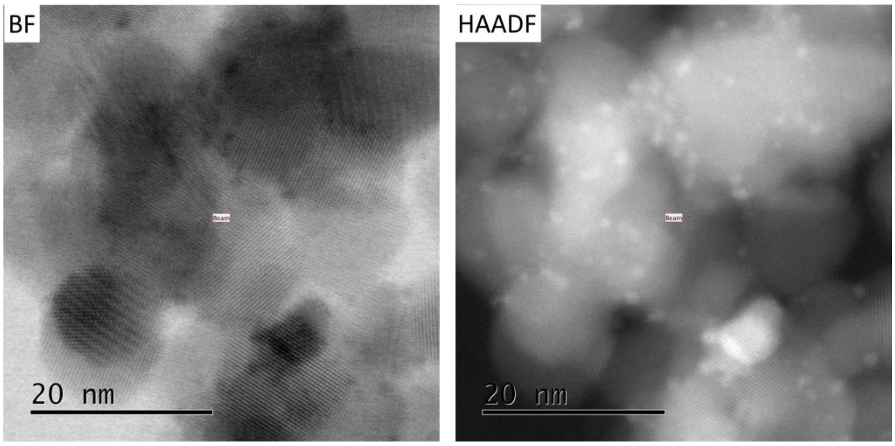

Figure S.6. **In situ STEM imaging.** Simultaneously acquired (a) bright field (BF) and (b) high angle annular dark field (HAADF) STEM imaging – illustrating that HAADF-STEM contrast provides better separation of nanoparticles from the support.

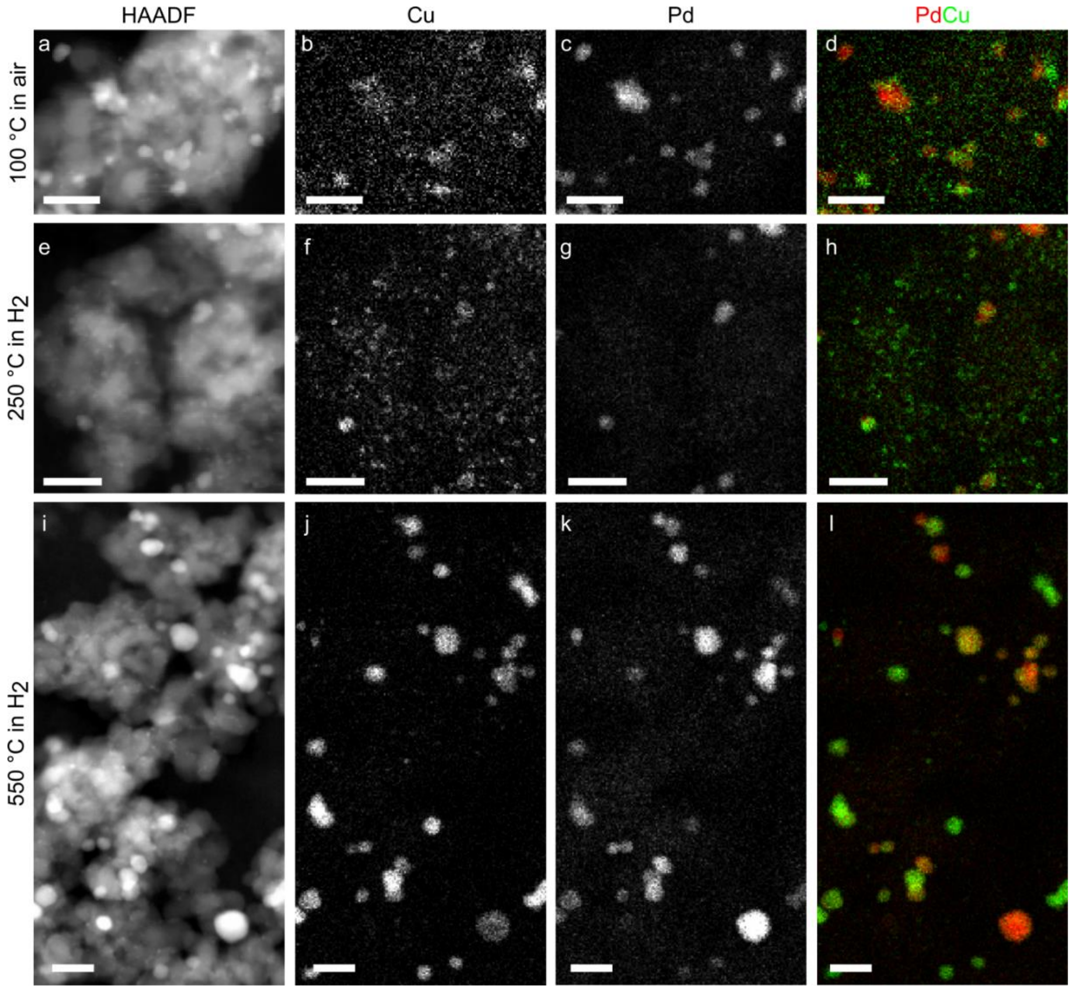

Figure S.7. In situ STEM-EDS mapping of changing elemental distributions. In situ STEM-HAADF (a, e, i) and STEM-EDS maps for Cu (b, f, j), Pd (c, g, k) and composite Pd and Cu (d, h, l) for the PdCu/TiO_2 catalyst under varying pre-treatment conditions (left column). Gas pressure is ~1 atm. Scale bars are 25 nm.

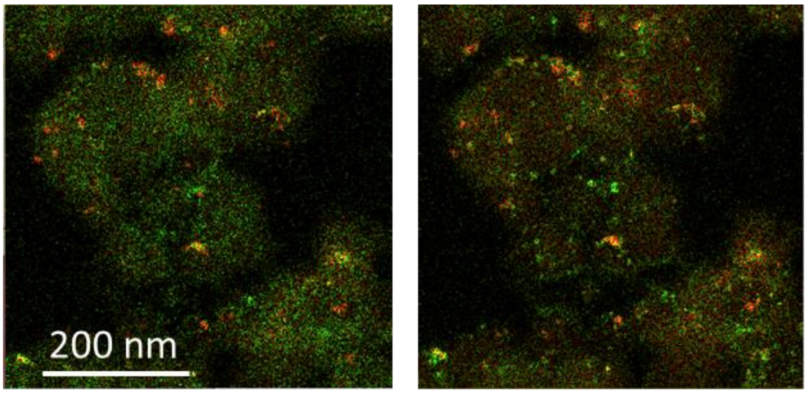

Figure S.8. In situ STEM-EDS mapping of changing elemental distributions measured on initial heating in situ in air at 100°C.

Figure 3 (main text) and Figure S.7 demonstrates the application of our modified STEM-EDS system to imaging our PdCu/TiO_2 catalyst under varying pre-treatment conditions. Figure S.7 includes the individual elemental maps for Pd and Cu used to produce the composite PdCu elemental maps shown and a larger field of view for Figure S.7 i-l.

We have chosen to illustrate different sample areas in Figure S.7 to minimise the risk of electron beam induced changes to the nanoparticle size and composition as a result of enhanced migration during the 2-5 minutes needed to acquire a suitable STEM-EDS elemental map. If the same area were imaged for each condition this would receive a high dose. Instead different areas were chosen and several regions of the sample compared to confirm the chosen areas were representative. It is possible to track dynamic changes to particle distribution within the same area by repeated spectrum imaging as illustrated in Figure S.8. However this was done at a reduced magnification in order to lower the electron dose required for each subsequent image.

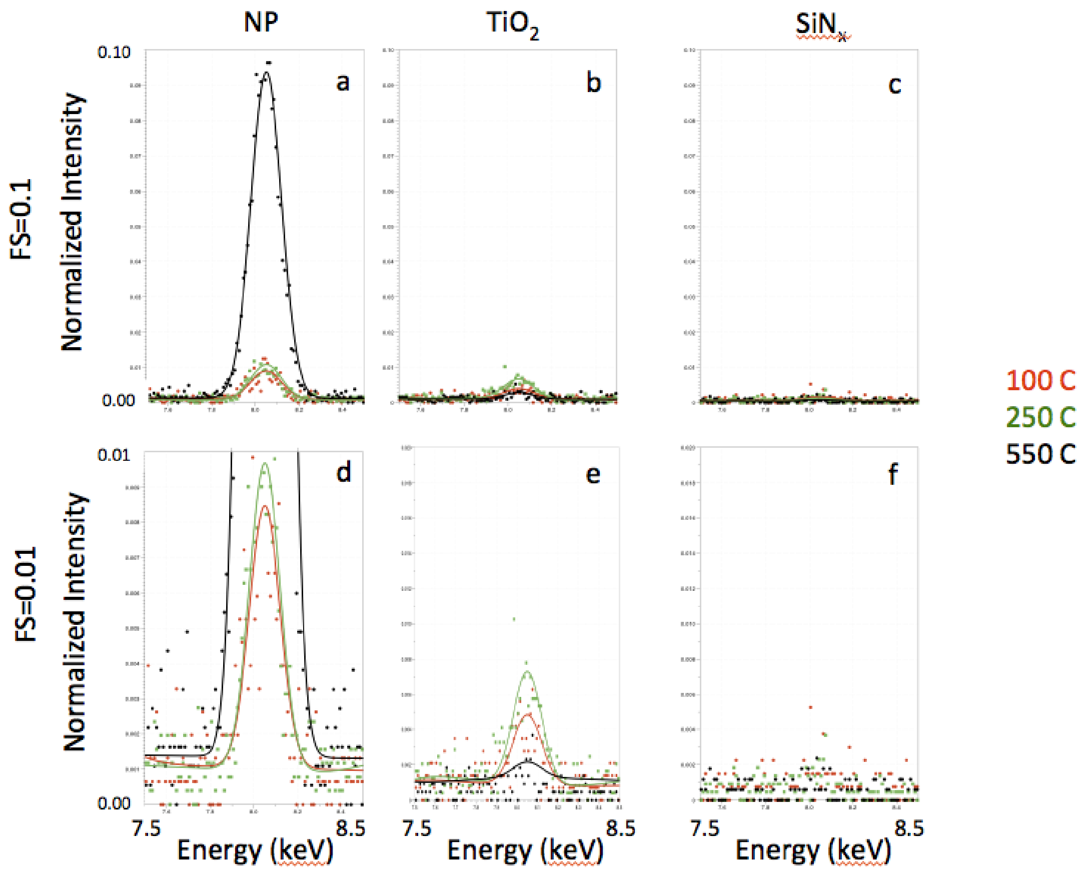

Figure S.9. STEM-EDS analysis showing the relative changes to the concentration of Cu metal during reduction. The signal from the nanoparticles (left column), is compared to that from the titania support regions without visible nanoparticles (middle column) and on a neighbouring region of the SiN window (right column). Peak shown is the Cu Kα peak at 8.04 keV. Spectra are colour-coded and refer to the same conditions shown in Figure S.6. Red = 100°C in air, Green = 250°C in hydrogen and, Black = 550°C in hydrogen. Upper and lower panels show the same data rescaled to more clearly illustrate different peak intensities. (a, b, c) = Full Intensity Scale (FS) = 0.1, (d, e, f) Full Intensity Scale (FS) = 0.01.

Using STEM-EDS data for the ratio of Cu metal signal normalized to the Si K peak from the SiN_x window of the e-Cell, it is possible to illustrate the relative change in concentration of the metal atoms in different regions of the catalyst. Figure S.9 shows examples of normalized spectra from selected nanoparticles (Figure S.9a, d), and the TiO_2 support (Figure S.9b, e), as well as the SiN_x window without any sample (Figure S.9c, f). The SiN_x window provides a reference of stray scattering due to the gas/windows but encouragingly this does not show any Cu signal at any stage of reduction.

During the reduction process the relative copper concentration (Figure S.9b, e) on the TiO_2 support rises slightly as nanoparticles begin to cluster and coalesce at 250°C then drops dramatically as larger NPs form, while the Pd content on the TiO_2 remains at background levels (not shown). As the nanoparticles grow in size with temperature their relative copper concentration increases most dramatically (Figure S.9a, d), while the Pd NPs concentration varies only by small amounts (not shown).

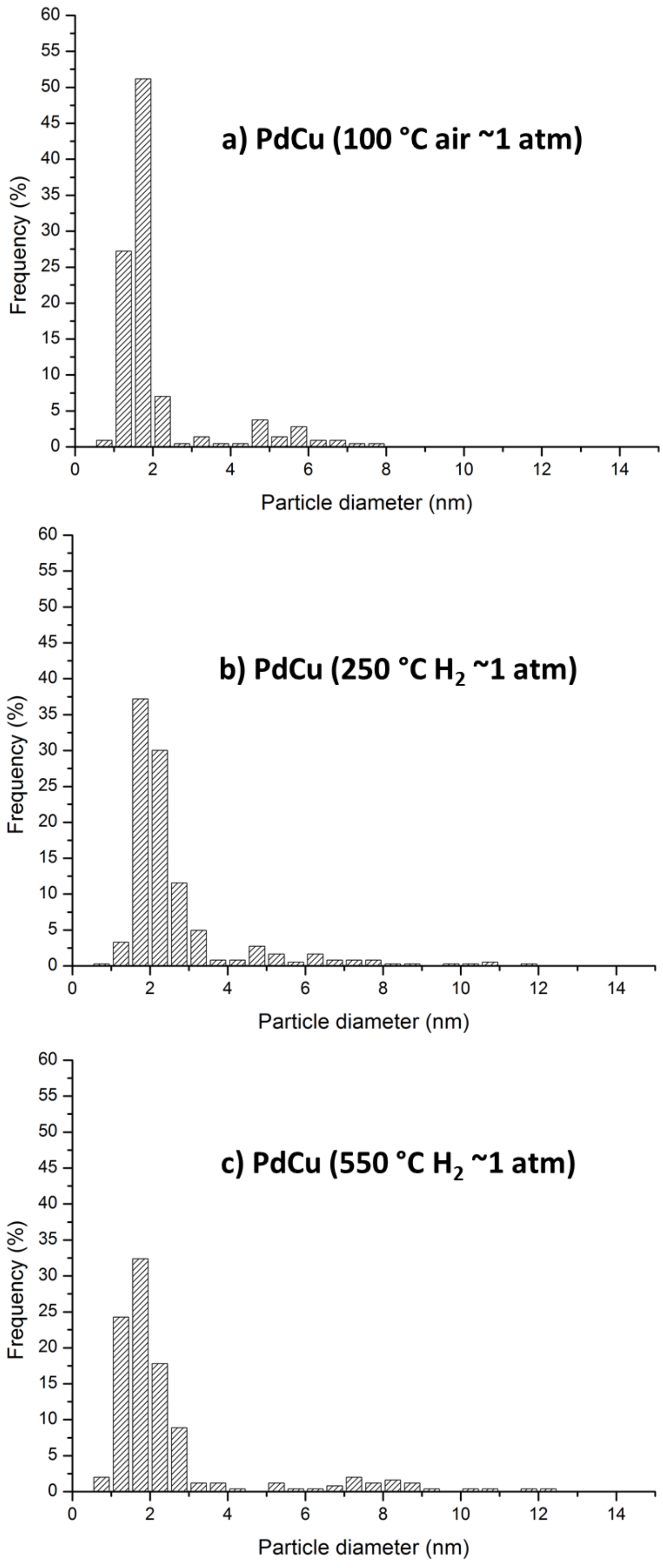

Figure S.10. **Quantitative analysis of the evolution of particle diameter as a function of in situ heating conditions.** Data was obtained from multiple different elemental maps and corresponds to the in situ conditions illustrated in Figure 3 of the main text. (a) calcined starting material in air at 100°C, (b) after heating in H_2, 1 atm, at 250°C, (c) after heating in H_2, 1 atm, at 550°C,

The in situ size analysis in Figure S.10 shows that there is a bimodal nanoparticle size distribution in the starting material after calcining with majority of the particles being ~1.5 nm in diameter. There is also a population of larger particles with diameters of ~5.5 nm. After heating in H_2, 1 atm, at 250°C the both particle types have increased in size slightly to ~2 nm and 6 nm respectively. After heating in H_2, 1 atm, at 550°C the smaller particles remain around 2nm in diameter but the larger particles have considerably increased in size to ~8 nm diameter.

Although the statistics are necessarily small (due to the time consuming nature of the experiments), this data illustrates the potential of this approach for obtaining quantitative data on particle size evolution in situ. The particles are highly heterogeneous in composition as illustrated in Figure 4, the relative composition of the catalyst nanoparticles are highly variable having a heterogeneous nature and thus determining an average composition of only a few particles will not be meaningful. Measuring a statistical distribution of NP compositional variations would be an interesting future extension of this work.

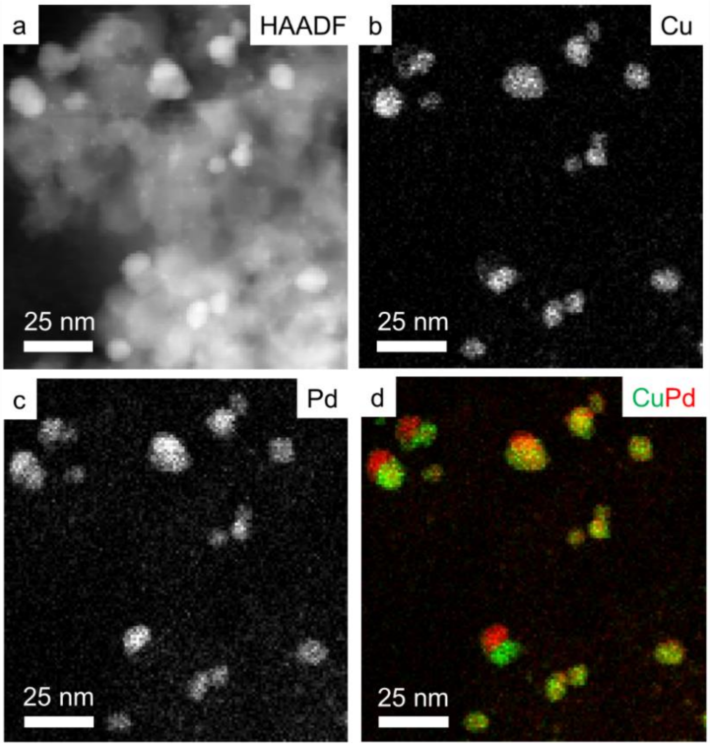

Figure S.11. Supporting information for Figure 4. (a) HAADF-STEM image (b) Cu, (c) Pd and (d) CuPd composite map. Image d is identical to that shown in Figure 4.

Figures S.11 – S.14 provide additional evidence of the different types of compositional segregation within the CuPd nanoparticles.

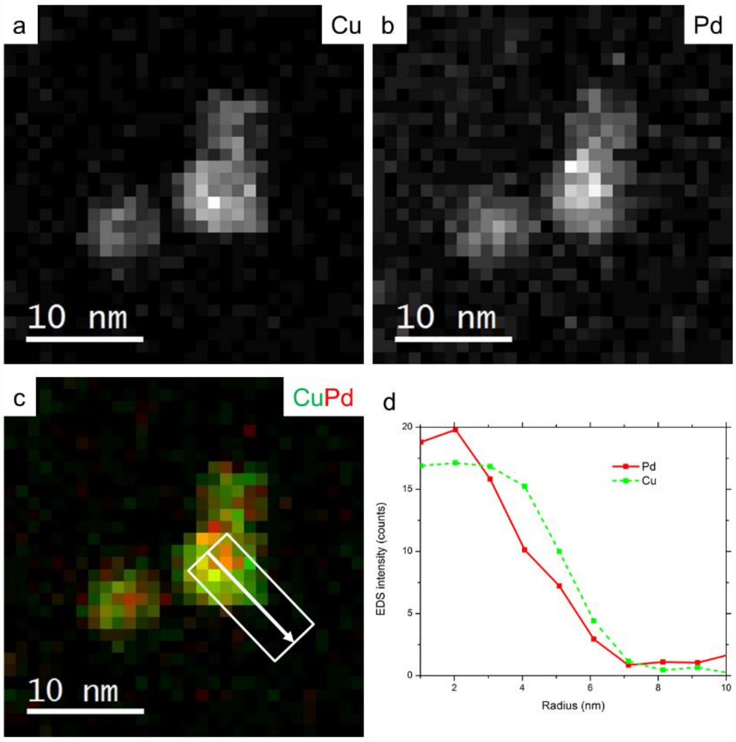

Figure S.12. Supporting information for Figure 4. (a) Cu, (b) Pd and (c) CuPd composite map and (d) line profile of the area marked in (c).

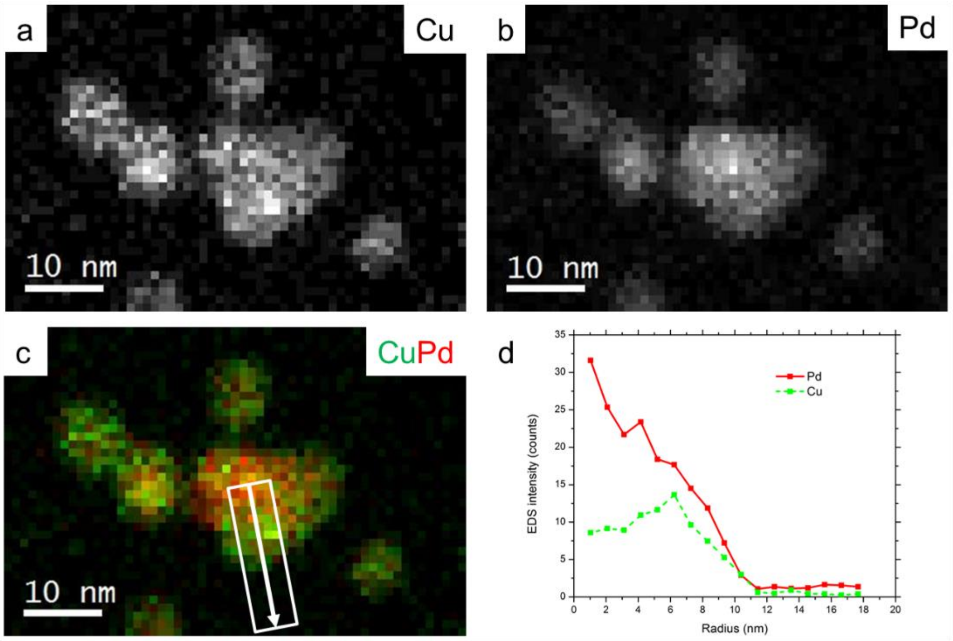

Figure S.13. Additional STEM-EDS data acquired after reduction at 550°C showing Cu-surface-rich NPs. (a) Cu, (b) Pd and (c) CuPd composite map and (d) line profile of the area marked in (c).

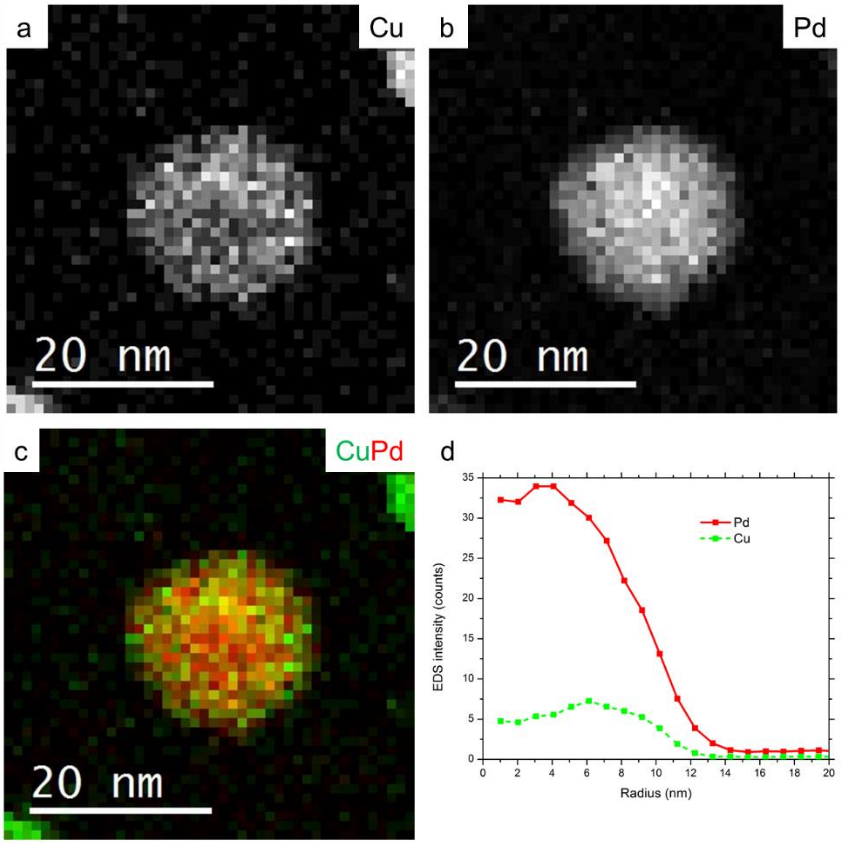

Figure S.14. Supporting STEM-EDS data acquired after reduction at 550°C showing Cu-surface-rich NPs. (a) Cu, (b) Pd and (c) CuPd composite map and (d) line profile of the area marked in (c).

SI REFERENCES

1. Batista, J., et al., *XPS and TPR examinations of γ-alumina-supported Pd-Cu catalysts.* Applied Catalysis A: General, 2001. **206**(1): p. 113-124.
2. Wada, K., et al., *Effect of supports on Pd–Cu bimetallic catalysts for nitrate and nitrite reduction in water.* Catalysis Today, 2012. **185**(1): p. 81-87.
3. Bazin, D. and J.J. Rehr, *Limits and Advantages of X-ray Absorption Near Edge Structure for Nanometer Scale Metallic Clusters*. The Journal of Physical Chemistry B, 2003. 107(45): p. 12398-12402.
